# Follicular dynamics and hormonal profile in Prepubertal nelore and Murrah heifers subjected to fixed-time artificial insemination (FTAI)

**DOI:** 10.1590/1984-3143-AR2025-0010

**Published:** 2026-04-10

**Authors:** Isabella de Oliveira Bêta, Larissa de Paiva Nunes Gonçalves, Aline Pacheco, Yana Eliza Feitosa de Almeida, Alisson Jordão Prado, Pietro Sampaio Baruselli, Kedson Alessandri Lobo Neves, Antonio Humberto Hamad Minervino

**Affiliations:** 1 Programa de Pós-graduação em Biociências, Universidade Federal do Oeste do Pará – UFOPA, Santarém, PA, Brasil; 2 Instituto de Biodiversidade e Florestas – IBEF, Universidade Federal do Oeste do Pará – UFOPA, Santarém, PA, Brasil; 3 Departamento de Reprodução Animal, Faculdade de Medicina Veterinária e Zootecnia, Universidade de São Paulo, São Paulo, SP, Brasil

**Keywords:** biotechnology, hormones, prepubertal, puberty, reproduction

## Abstract

We aimed to evaluate puberty induction, follicular dynamics, and hormonal profiles of progesterone (P4) and estradiol (E2) in prepubertal Nelore and Murrah heifers subjected to synchronization for fixed-time artificial insemination (FTAI). Six Nelore heifers (14 to 17 months old; 320 to 350 kg) and six Murrah heifers (14 to 17 months old; 422 to 523 kg) confirmed as prepubertal by ultrasonography due to the absence of a corpus luteum were used. The protocol included an intravaginal P4 device (0.5 mg) on D0, along with the administration of estradiol benzoate (EB) and prostaglandin F2α. In Nelore, the device was removed on D7, followed by the administration of estradiol cypionate, prostaglandin F2α, and eCG, with insemination occurring 48–56 hours after intravaginal device removal (D9). In Murrah, the device was removed on D9, followed by prostaglandin F2α and Ecg administration, with insemination on D12 after GnRH administration (D11). Blood samples were collected (Nelore: D7 and D9; Murrah: D9 and D12) to measure hormone levels, and ultrasonography monitored follicular dynamics and ovulation. The ovulatory follicle diameter was 7.83 ± 2.25 mm (Nelore) and 7.7 ± 1.63 mm (Murrah), with no significant differences (P > 0.05). The dominant follicle diameter at P4 device removal was 6.5 ± 1.59 mm (Nelore) and 6.9 ± 4.97 mm (Murrah). The mean P4 levels were 0.76 ng/mL (Nelore) and 1.42 ng/mL (Murrah) at P4 device removal, and 1.00 ng/mL and 0.52 ng/mL at the moment of FTAI. The mean E2 levels were 9.28 pg/mL (Nelore) and 26.75 pg/mL (Murrah) at the P4 device removal, and 11.20 pg/mL and 11.59 pg/mL at the moment of FTAI. Ovulation rates were 100% in Nelore and 50% in Murrah. These results highlight the importance of tailoring FTAI protocols to the specific reproductive characteristics of each breed.

## Introduction

Brazil stands out as the largest exporter and the largest producer of beef in the world, with a herd of approximately 238 million head (IBGE, 2024). The North region has the second-largest cattle herd in the country, and Pará leads in buffalo farming, with over 750,000 buffaloes (ADEPARÁ, 2023). This prominence results from advanced natural conditions and technological progress, establishing livestock farming as a strategic sector for the country's economy (ABIEC, 2024).

Puberty in heifers is influenced by genetic, nutritional, and environmental factors. Nelore heifers, representing Zebu cattle in Brazil, reach puberty between two and three years old, with genetic and nutritional improvements enabling anticipation to 14 months in intensive systems (Ferraz et al., 2018). In contrast, Murrah buffaloes exhibit late sexual maturity, typically between 24 and 30 months, due to lower estrus expression and reproductive seasonality (Neglia et al., 2020). These factors directly impact the efficiency of production systems (Baruselli et al., 2022; Hafez, 1954; Zicarelli and Vale, 2002). FTAI is a widely used biotechnology in livestock farming, allowing for ovulation synchronization and optimizing reproductive rates (Baruselli et al., 2021). In cattle, hormonal protocols based on estrogen and progesterone achieve pregnancy rates exceeding 50% (Silva et al., 2022). In buffaloes, specific protocol adjustments, such as the inclusion of eCG, is necessary due to the reproductive peculiarities of the species, ensuring advances in production efficiency and sustainable herd management (Ribeiro, 2023).

This study aimed to induce puberty, evaluate follicular dynamics, and analyze the hormonal profiles of prepubertal Nelore and Murrah heifers subjected to FTAI. Based on follicular monitoring and ovulation scheduling, the research seeks to contribute to improving reproductive practices, promoting greater efficiency and profitability in animal production, especially in differentiated management systems for cattle and buffaloes.

## Methods

This experiment was approved by the Ethics Committee for the Use of Animal of the Federal University of Western Pará (CEUA/UFOPA), under protocol no. 0520230262.

### Experimental location, animals, and management

The experiments were conducted on farms located in the rural area of the municipality of Mojuí dos Campos, Pará, Brazil, specifically on the Santo Antônio (-2.6771, -54.6260) and Jaraguá (-2.7759, -54.4374) farms, during February and March 2023.

### Experimental design and hormonal protocols

#### Experiment 1

The experiment involved six Nelore heifers, all prepubertal, with an average age of 14 ± 17 months. The heifers weighed between 320 and 350 kg. Prepubertal status was confirmed through transrectal ultrasound, which verified the absence of a corpus luteum and evaluated ovarian and uterine thickness. During the experiment, the heifers were kept grazing on *Brachiaria brizantha* cv. Marandu, with mineral salt supplementation and ad libitum access to water.

The heifers underwent a fixed-time artificial insemination (FTAI) protocol. On the initial day (D0), an intravaginal device containing 0.5 mg of progesterone (*DIB®*, Zoetis, Brazil) was inserted, and 2 mg of estradiol benzoate (*BE*; *Bioestrogen®*, Biogénesis Bagó, Brazil) and 5 mg of dinoprost trometamina (*Lutalyse®*, Zoetis, Brazil) were administered intramuscularly (IM).

**Figure 1:**
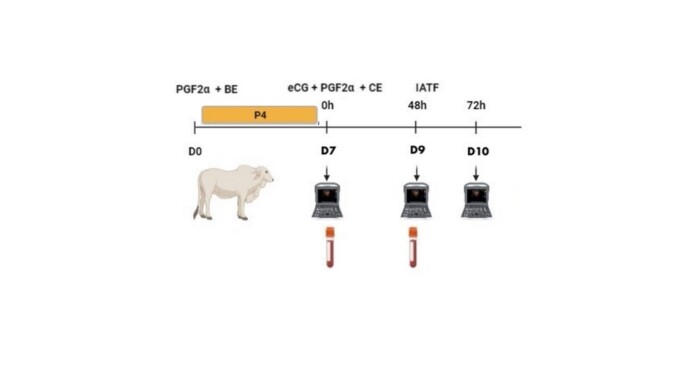
Flowchart of the fixed-time artificial insemination (FTAI) protocol in bovine females. The protocol includes steps such as hormone administration (PGF2α, BE, eCG, and CE), ultrasonography to monitor follicular dynamics on days D7, D9, and D10, and blood collection for hormone assays.

On D7, the intravaginal device was removed, and 0.5 mg of estradiol cypionate (*ECP®*, Zoetis, Brazil), 12.5 mg of dinoprost trometamina, and 300 IU of eCG (*Novormon®*), Zoetis, Brazil) were administered. The heifers were inseminated on D9, between 48 and 56 hours after the device's removal. The methods used are illustrated in the flowchart shown in Figure 1.

#### Experiment 2

The experiment involved six Murrah heifers, with an average age of 14 ± 17 months and weighing between 422 and 523 kg. Prepubertal status was confirmed through transrectal ultrasound to verify the absence of a corpus luteum and to evaluate ovarian and uterine thickness. During the experiment, the animals were kept grazing on *Brachiaria brizantha* cv. Marandu, with mineral salt supplementation and ad libitum access to water.

**Figure 2:**
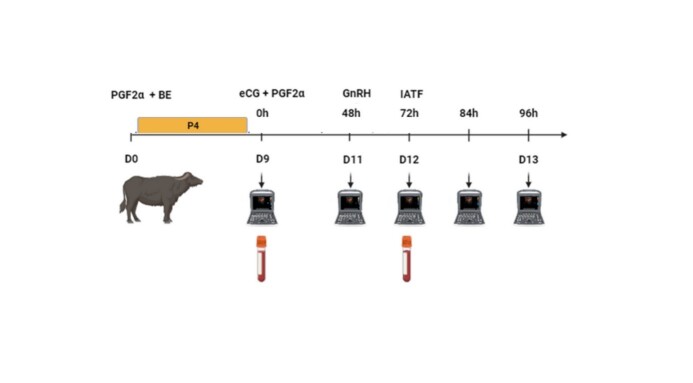
Flowchart of the fixed-time artificial insemination (FTAI) protocol in buffaloes. The diagram includes sequential hormone administration (PGF2α, BE, eCG, and GnRH), follicular dynamics monitoring by ultrasonography on days D9, D11, D12, and D13, and blood collection for hormonal evaluation.

The heifers were subjected to a fixed-time artificial insemination (FTAI) protocol. On D0, an intravaginal device containing 0.5 mg of progesterone was inserted, and 2 mg of estradiol benzoate (BE) and 5 mg of dinoprost trometamina (*Lutalyse®*, Zoetis, Brazil) were administered intramuscularly (IM). On D9, the device was removed, followed by the administration of 12.5 mg of dinoprost trometamina and 400 IU of eCG. On D11, 2.5 mg of GnRH (*Tec-relin®*, Tecnopec) was administered at 4:00 PM, and insemination was performed on D12 at 8:00 AM. The methods used are illustrated in the flowchart shown in Figure 2.

### Follicular dynamics

The ovaries were evaluated by transrectal ultrasound (Mindray DP-50Vet, 5 MHz linear transducer) on D0, D7, D9, and D10 (Experiment 1) and on D0, D9, D11, D12, and D13 (Experiment 2). Ovulation was monitored every 12 hours after insemination until the disappearance of the ovulatory follicle was confirmed.

#### Blood collection and plasma concentrations of P4 and E2

Blood samples were collected via jugular venipuncture on D8 and D10 (Experiment 1) and on D9 and D11 (Experiment 2). Samples were collected in tubes containing a clot activator. Plasma was separated by centrifugation and stored at -20°C.

Concentrations of P4 and E2 were determined using chemiluminescence in an outsourced laboratory to assess their relationship with follicular diameter.

### Statistical analysis

The data were organized in Microsoft Excel and analyzed using RStudio. Normality (Shapiro-Wilk test) and homogeneity of variances (Levene's test) were verified. In cases of assumption violations, nonparametric tests (Wilcoxon or Friedman) were applied. Linear and polynomial regressions were also performed to explore the relationships between hormonal levels and follicular dynamics.

The statistical analysis results revealed significant insights into follicular dynamics and the hormonal levels of progesterone (P4) and estradiol (E2) in Nelore and Murrah heifers subjected to FTAI protocols. Regarding follicular dynamics, the paired t-test in Nelore indicated a numerical increase in follicular diameter from D7 to D9 (mean difference = 1.33 mm), but without statistical significance (p = 0.065). For Murrah, the violation of normality on D9 required the application of the Wilcoxon test, which also revealed no statistically significant differences between D9 and D11 (p = 0.304).

In terms of hormones, P4 levels in Murrah showed greater variability and a significant drop on D12 (0.52 ± 0.31 ng/mL), attributed to PGF2α-induced luteolysis. In contrast, Nelore exhibited a consistent increase from D7 (0.76 ± 0.32 ng/mL) to D10 (1.00± 0.61 ng/mL), reflecting corpus luteum functionality. For E2, linear regression analysis demonstrated a moderate positive correlation with follicular diameter (R^2^ = 0.57), highlighting estradiol's role in follicular growth, although E2 levels in Murrah showed greater variability.

Polynomial regression in Nelore revealed a nonlinear relationship between P4 and E2 (R^2^ = 0.55), whereas in Murrah, the correlation between the hormones was virtually nonexistent (R^2^ = 0.0008). Despite the lack of significant differences in some analyses, the results reflect hormonal patterns consistent with the literature, emphasizing the effectiveness of the protocols used. However, they also underscore physiological differences between the species that could influence reproductive outcomes.

## Results

### Experiment 1

The mean diameters of the dominant follicle (DFD) and the ovulatory follicle (DFOL) in Nelore heifers were 6.5 ± 1.59 mm and 7.83 ± 2.25 mm, respectively. A comparison of DFD and DFOL on days 7 (D7) and 9 (D9) revealed normality (Shapiro- Wilk, p > 0.05) and homogeneity of variances (Levene, p > 0.05), allowing the use of the paired t-test. Although a numerical increase in follicular diameter from D7 to D9 was observed (mean difference = 1.33 mm), this increase was not statistically significant (t(5) = 2.35; p = 0.065), possibly due to the limited sample size (N=6).

The critical interval between 48 and 72 hours after P4 device removal and estradiol cypionate administration was identified as essential for the success of synchronized ovulation, with all Nelore heifers ovulating 24 hours after FTAI, resulting in a 100% ovulation rate, as shown in Table 1.

**Table 1:** Reproductive parameters of Nelore heifers subjected to a fixed-time artificial insemination (FTAI) protocol. Includes the mean diameter of the dominant follicle at the time of progesterone (P4) device removal, the mean diameter of the ovulatory follicle (DFOL), and the ovulation rate (%).

**PARAMETERS**	**NELORE**
Dominant follicle diameter at P4 device removal	6.5±1.59mm a
Ovulatory follicle diameter (DFOL)	7.83±2.25mm a
Ovulation rate (%)	100% (6/6)

Means followed by the same letter did not vary statistically (*P* > 0.05).

Hormonal levels displayed consistent patterns throughout the FTAI protocol. The mean progesterone (P4) concentration was 0.76 ± 0.32 ng/mL on D7 and increased to 1.00 ± 0.61 ng/mL on D10.Estradiol (E2) levels were 9.28 ± 0.69 pg/mL on D7 and increased to 11.20 ± 2.30 pg/mL on D10.

The relationship between hormonal levels and follicular dynamics showed a weak negative correlation between P4 and follicular diameter (R^2^ = 0.04), while the correlation between E2 and follicular diameter was moderate (R^2^ = 0.57). Polynomial regression analysis indicated that the relationship between E2 and P4 accounted for 55% of hormonal variability (R^2^ = 0.55), demonstrating an initial increase in E2 with rising P4 levels, followed by stabilization, as represented in Table 2

**Table 2:** Hormone concentrations of progesterone (P4) in Nelore and Murrah heifers during the fixed-time artificial insemination (FTAI) protocol. Hormone concentrations were measured on D7 and D10 for Nelore and on D9 and D12 for Murrah.

**DAY**	**HORMONE**	**NELORE**	**DAY**	**HORMONE**	**MURRAH**
D7	P4	0.76± 0.32ng /mL	D9	P4	1.42±1.56 ng/mL
D10	P4	1.00 ±0.61 ng/mL	D12	P4	0.52± 0.31ng/mL

Values are presented as mean ± standard deviation.

### Experiment 2

The mean diameters of the dominant follicle (DFD) and the ovulatory follicle (DFOL) in Murrah buffaloes were 6.9 ± 4.97 mm and 7.7 ± 1.63 mm, respectively. The analysis of follicular dynamics demonstrated a violation of normality on D9 (W = 0.700, p = 0.006), but normality was observed on D11 (W = 0.867, p = 0.257). A comparison between D9 and D11 using the Wilcoxon rank-sum test with continuity correction showed no statistically significant differences in follicular diameters (W = 9, p = 0.304).

The ovulation rate was 50%, with one buffalo ovulating on the day of FTAI (D11), 48 hours after the administration of P4 device removal + PGF2α + eCG, and two others ovulating on D12 one in the morning (24 hours after FTAI) and the other in the afternoon (36 hours after FTAI), as represented in Table 3.

**Table 3:** Reproductive parameters of Murrah buffaloes subjected to a fixed-time artificial insemination (FTAI) protocol. Includes the mean diameter of the dominant follicle at the time of progesterone (P4) device removal, the mean diameter of the ovulatory follicle (DFOL), and the ovulation rate (%). Means followed by the same letter did not differ statistically (*P* > 0.05).

**PARAMETERS**	**MURRAH**
Dominant follicle diameter at P4 device removal	6.9 ± 4.97 mm a
Ovulatory follicle diameter (DFOL)	7.7 ± 1.63 mm a
Ovulation rate (%)	50% (3/6)

Means followed by the same letter did not differ statistically (P > 0.05).

Hormonal levels showed greater variability compared to Nelore. The mean progesterone (P4) concentration was 1.42 ± 1.56 ng/mL on D9, decreasing to 0.52 ± 0.31 ng/mL on D12, reflecting the removal of the P4 intravaginal device. Estradiol (E2) levels were 26.75 ± 41.13 pg/mL on D9, decreasing to 11.59 ± 6.35 pg/mL on D12. The high variability in E2 levels on D9, evidenced by the elevated standard deviation, may be attributed to the physiological characteristics of buffaloes, such as higher hormonal sensitivity and a greater number of follicular waves per cycle.

The relationship between P4 and follicular dynamics showed an extremely weak correlation (R^2^ = 0.0008), while E2 demonstrated a more pronounced pattern of influence on follicular dynamics, as represented in Table 4.

**Table 4:** Hormone concentrations of estradiol (E2) in Nelore and Murrah heifers during the fixed-time artificial insemination (FTAI) protocol. Hormone concentrations were measured on D7 and D10 for Nelore and on D9 and D12 for Murrah.

**DAY**	**HORMONE**	**NELORE**	**DAY**	**HORMONE**	**MURRAH**
D7	E2	9.28 ± 0.69 pg/mL	D9	E2	26.75 ± 41.13 pg/mL
D10	E2	11.20 ± 2.30 pg/mL	D12	E2	11.59 ± 6.35 pg/mL

Values are presented as mean ± standard deviation.

## Discussion

The data obtained in this study highlight significant physiological differences between prepubertal Nelore (*Bos indicus*) and Murrah (*Bubalus bubalis*) heifers subjected to fixed-time artificial insemination (FTAI) synchronization protocols, particularly regarding follicular dynamics and hormonal profiles. These differences emphasize the need for specific protocols adapted to the unique characteristics of each species and physiological stage.

As observed, the ovulation rate was higher in Nelore heifers compared to Murrah heifers, which may be related to differences in circulating progesterone concentrations, hypothalamic-pituitary responsiveness, and the population of antral follicles. Baldrighi et al. (2022) reported that buffaloes have lower plasma progesterone concentrations and smaller corpus luteum volumes, which may compromise reproductive efficiency in this species. In contrast*, Bos indicus* cattle exhibit a greater number of follicular waves and a larger population of antral follicles, which favors follicular selection and responsiveness to exogenous hormones.

Additionally, the study by Silva et al. (2022) demonstrated that the use of eCG in Nelore heifers resulted in higher ovulation and pregnancy rates compared to the use of FSH, highlighting the importance of hormone selection in FTAI protocols, especially in animals raised under extensive systems and tropical conditions. Similar results were reported by Sá et al. (2010), in which the inclusion of eCG in fixed-time artificial insemination protocols for Nelore heifers resulted in ovulation rates exceeding 90%, along with increased follicular diameter, enhanced corpus luteum development, and improved pregnancy rates.

In buffaloes, treatment with eCG at the time of device removal increases the diameter of the dominant follicle at the time of TAI and the ovulation rate; during the subsequent diestrus, the treatment results in an increased corpus luteum (CL) diameter, elevated progesterone (P4) concentrations, and enhanced luteal function (Carvalho et al., 2013).

Although the present study used a standardized protocol for both species, the responses differed, suggesting that adjustments such as the increased doses of eCG may be particularly beneficial for buffaloes.

The difference in dominant follicle diameter and estradiol production may also be explained by the effect of circulating progesterone concentrations throughout the protocol. According to Silva et al. (2025), higher circulating progesterone concentrations, achieved with new intravaginal devices, delayed follicular deviation and resulted in smaller dominant follicles with reduced estradiol production. These findings suggest that such effects may be particularly relevant for prepubertal Murrah heifers, whose endogenous endocrine responsiveness may be less efficient.

It is important to emphasize that interspecies differences go beyond ovarian physiology. Factors such as metabolism, IGF-1 and insulin secretion, and age at puberty directly influence the response to reproductive protocols (Baldrighi et al., 2022).

Understanding these particularities is essential for improving biotechnologies applied to the reproduction of cattle and buffaloes, especially in FTAI programs with prepubertal heifers. Furthermore, studies like that of Silva et al. (2022) indicate that under tropical conditions and extensive systems, the use of long-acting hormones, such as eCG, may be more advantageous in ensuring greater follicular synchronization and improved pregnancy rates.

### Experiment 1

Moreover, the DFOL values obtained in Nelore heifers align with those of Lemes (2018), who reported diameters of 6.4 mm and 6.7 mm in animals treated with intravaginal and injectable progesterone. Gimenes et al. (2008) observed that ovulatory capacity is acquired when the dominant follicle reaches between 7.0 and 8.4 mm in diameter in Bos taurus indicus heifers and increases significantly when the follicle reaches 8.5 mm in diameter, corroborating the 7.83 ± 2.25 mm found in this study. These findings confirm that the protocol used effectively facilitated follicular maturation, enabling the development of follicles within the functional threshold for ovulation.

The hormonal observed behavior also reinforces the existing literature. Progesterone (P4) levels increased from 0.76 ± 0.32 ng/mL on D7 to 1.00 ± 0.61 ng/mL on D10, which is consistent with previous studies demonstrating a progressive rise in plasma progesterone concentrations following ovulation, associated with corpus luteum formation and early luteal development in heifers submitted to progesterone-based synchronization protocols (Mantovani et al., 2010). Additionally, estradiol (E2) levels, which rose from 9.28 ± 0.69 pg/mL on D7 to 11.20 ± 2.30 pg/mL on D10, follow the pattern described by Sousa (2017), who noted progressive increases in E2 produced by the dominant follicle in prepubertal heifers treated with progesterone devices.

The 100% ovulation rate in Nelore heifers, with ovulation occurring 24 hours after insemination and 72 hours after the administration of EC + PGF2α + eCG. These results, combined with the uniformity observed in follicular and hormonal growth patterns, demonstrates the robustness of the protocol used in promoting efficient synchronization and consistent reproductive outcomes. In contrast, Araújo et al. (2019) reported an ovulation rate of 82.22% in prepubertal heifers, indicating that reproductive maturity may influence the ovulatory response to hormonal protocols.

### Experiment 2

The results obtained for Murrah buffaloes in this study highlight the challenges and specificities of hormonal and follicular dynamics in this species. The mean diameter of the dominant follicle (DFD) of 6.9 ± 4.97 mm and the ovulatory follicle (DFOL) of 7.7 ± 1.63 mm align with the values reported by Rautela et al. (2024), who observed average follicular diameters of approximately 7 mm in Murrah buffaloes treated with hormonal protocols. This similarity underscores the consistency of hormonal protocols in promoting adequate follicular growth in buffaloes as well.

However, the greater variability observed in the results for Murrah reflects the hormonal particularities of the species. The violation of normality in the data on D9 (W= 0.700, p = 0.006) and the lack of statistically significant differences between follicular diameters on D9 and D11 (W = 9, p = 0.304) support the findings of Baruselli et al. (2007), who highlighted the heightened sensitivity of the hypothalamic-pituitary axis in buffaloes.

Studies by Porto-Filho (2000) and Kumar et al. (1991) also confirmed greater variability in follicular and hormonal parameters in buffaloes, particularly compared to Zebu cattle.

The 50% ovulation rate observed in this study aligns with reports by Sales et al. (2012) and Rautela et al. (2024), who observed similar rates in Nellore and Murrah, emphasizing the impact of factors such as seasonality and thermal stress on reproductive performance. The variation in ovulation timing, with intervals between 24 and 36 hours post-insemination, is consistent with the ovulation windows of 48 to 96 hours described in the literature, highlighting the need for protocol adjustments to optimize synchronization.

The hormonal levels observed in Murrah confirm previously described patterns. Progesterone (P4) levels decreased from 1.42 ± 1.56 ng/mL on D9 to 0.52 ± 0.31 ng/mL on D12, consistent with findings by Ginther et al. (2007), who reported similar declines associated with PGF2α-induced luteolysis. Elevated estradiol (E2) levels on D9 (26.75 ± 41.13 pg/mL), followed by a reduction on D12 (11.59 ± 6.35 pg/mL), corroborate the patterns described by Taponen et al. (1999), who observed similar hormonal fluctuations in buffaloes subjected to hormonal protocols.

The findings of this study, in line with the literature, reinforce that hormonal protocols can promote follicular growth and ovulation in Murrah buffaloes. However, the greater variability observed compared to Nelore heifers suggests the need for specific adjustments, such as modulation of eCG dosage or alterations in the progesterone device exposure time, as suggested by Baruselli et al. (2007).

## Conclusion

Fixed-time artificial insemination (FTAI) proved to be an effective tool for inducing puberty and controlling follicular dynamics in prepubertal Nelore and Murrah heifers. The protocol promoted uniform follicular development, evidenced by the absence of significant differences in follicular diameters between the species, demonstrating its ability to consistently stimulate ovarian cyclic activity.

The analysis of hormonal profiles revealed differences in progesterone and estradiol levels between Nelore and Murrah, reflecting species-specific physiological particularities. These findings highlight the need for tailored reproductive protocols that meet the biological demands of different breeds and emphasize the importance of adapting strategies to maximize FTAI efficiency.

This research not only achieved its proposed objectives but also underscored the essential role of reproductive biotechnology in improving the productivity and sustainability of livestock farming. Thus, this work contributes to understanding the reproductive particularities of cattle and buffaloes, providing a solid scientific foundation for improving reproductive management strategies and promoting more efficient and sustainable livestock systems.

## Data Availability

Research data is available in the body of the article.
